# Pyrazinamide Resistance, *Mycobacterium tuberculosis* Lineage and Treatment Outcomes in San Francisco, California

**DOI:** 10.1371/journal.pone.0095645

**Published:** 2014-04-23

**Authors:** Jonathan M. Budzik, Leah G. Jarlsberg, Julie Higashi, Jennifer Grinsdale, Phil C. Hopewell, Midori Kato-Maeda, Payam Nahid

**Affiliations:** 1 Department of Medicine, University of California San Francisco, San Francisco, California, United States of America; 2 Tuberculosis Control Unit, San Francisco Department of Public Health, San Francisco, California, United States of America; 3 Division of Pulmonary and Critical Care Medicine, Curry International Tuberculosis Center, San Francisco General Hospital, University of California San Francisco, San Francisco, California, United States of America; St. Petersburg Pasteur Institute, Russian Federation

## Abstract

**Background:**

Pyrazinamide (PZA) is a first line agent for the treatment of active tuberculosis. PZA is also considered a potent companion drug for newer regimens under development. There are limited data on the demographic, clinical, and pathogen characteristics of PZA resistant tuberculosis.

**Methods:**

Using a retrospective cohort study design, we evaluated all PZA resistant *M. tuberculosis* (*M.tb*) and *M. bovis* cases reported in San Francisco from 1991 to 2011. Demographic, clinical, and molecular data were analyzed. *M.tb* lineage was determined for all PZA resistant strains and compared to PZA susceptible strains.

**Results:**

PZA resistance was identified in 1.8% (50 of 2,842) of mycobacterial isolates tested, corresponding to a case rate of 0.3 per 100,000 in the population. Monoresistant PZA infection was associated with the Hispanic population ([OR], 6.3; 95% [CI], 1.97–20.16) and 48% of cases were due to *M. bovis*. Infection with monoresistant PZA was also associated with extrapulmonary disease ([OR], 6.0; 95% [CI], 2.70–13.26). There was no statistically significant difference between treatment failure and mortality rates in patients infected with PZA monoresistance compared to pansusceptible controls (4% vs. 8%, *p* = 0.51), or those with PZA and MDR resistance (PZA-MDR) compared to MDR controls (18% vs. 29%, *p* = 0.40). PZA resistance was not associated with *M.tb* lineage.

**Conclusions:**

Across two decades of comprehensive epidemiologic data on tuberculosis in San Francisco County, PZA resistance was uncommon. PZA resistance caused predominantly extrapulmonary disease and was more common in Hispanics compared to other ethnicities, with nearly half the cases attributed to *M. bovis*. No association was found between PZA monoresistance and *M.tb* lineage. Treatment outcomes were not adversely influenced by the presence of PZA resistance.

## Introduction

Pyrazinamide (PZA) monoresistance incidence among *M.tb* complex cases, as reported by the US National TB Surveillance System (NTSS), is 2.0–3.3% and increasing over time [1]. Moreover, 38% of MDR cases also harbor resistance to PZA, which results from inactivating mutations in the *pnc*A allele [1], [2]. In a retrospective cohort study spanning 11 years, cases in Quebec infected with PZA monoresistant *M.tb* were less likely to be categorized as cured after treatment compared to patients infected with pan-susceptible *M.tb* (adjusted odds ratio [aOR], 0.4; 95% confidence interval [CI], 0.2–0.8) [3]. An improved understanding of PZA monoresistance and its influence on treatment response in other regions is needed as new duration shortened regimens are under development that include PZA as a companion drug [4].

*M.tb* strains are classified into seven lineages based on geography and genetic polymorphism [5], [6]. The Quebec study did not have lineage information; however, PZA resistance rates differed among tuberculosis lineages in isolates from the NTSS 10 year study involving 38 jurisdictions in the USA [1]. The Indo-Oceanic lineage was associated with 2.3 fold increase in the adjusted prevalence ratio (aPR) of PZA resistance compared to the East Asian lineage, but in a MDR background the PZA resistance rate was decreased by 0.54 fold [1]. However, the investigators did not determine the impact of PZA resistance on treatment response.

In the present study, we determined the incidence of PZA resistance from all patients reported to the City and County of San Francisco Tuberculosis Control Clinic using 21 years of comprehensive molecular epidemiology data. We analyzed the impact of PZA resistance, including the inherently PZA resistant organism *M. bovis* [7], on treatment outcomes specifically in comparison to otherwise drug-susceptible *M.tb* and MDR *M.tb*. We also assessed the association between PZA resistance and bacterial lineage.

## Methods

### Ethics statement

This study named “Molecular Epidemiology of Tuberculosis and Contact Investigation” was approved by the San Francisco Department of Public Health (SFDPH) and the UCSF Human Research Protection Program Institutional Review Board (approval #10-00937 and #10-00925). Patient records and information were collected as part of routine surveillance for public health. Patient material was anonymized and de-identified prior to analysis. As a consequence, written informed patient consent for participation in the research study was not obtained.

### Study design

**Figure 1:**
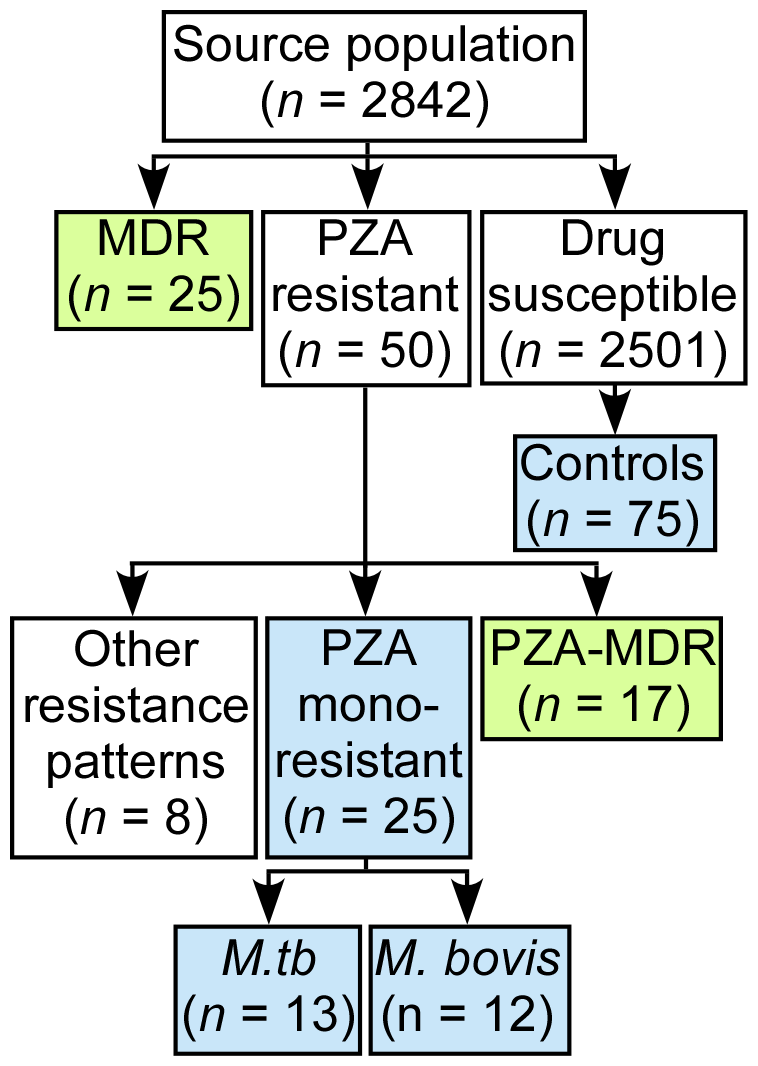
Flow diagram of cohort selection. Between 1991–2011 in San Francisco county, there were 2842 cases of culture confirmed tuberculosis infection with complete antibiotic susceptibility data. Among these, 25 MDR cases and 50 PZA resistant cases were reported. Three time-matched drug susceptible controls (*n* = 75) with the same site of infection, pulmonary or extrapulmonary, were selected among the total number of susceptible controls (*n* = 2501) for each PZA monoresistant case. The blue boxes indicate the first case-control group, which compared patients infected with drug susceptible *M.tb* (*n* = 75) versus PZA monoresistant *M.tb* (*n* = 13) or *M. bovis* (*n* = 12). The green boxes indicate the second case-control group, which compared patients infected with MDR (*n* = 25) versus PZA-MDR (*n* = 17) *M.tb*. There were eight cases of infection with PZA resistance patterns that did not meet criteria for PZA monoresistance or PZA-MDR.

Between January 1991 and December 2011, the records of the SFDPH Tuberculosis Control Section were reviewed to identify culture confirmed tuberculosis cases with available antibiotic susceptibility data (Figure 1). In this retrospective cohort study, patients within two case-control groups were compared in order to determine the impact of PZA resistance on outcomes. The first case-control group compared patients infected with PZA monoresistant *M. tuberculosis* or *M. bovis* and pansusceptible *M.tb* (Figure 1). Three next available time-matched antibiotic susceptible controls (*n* = 75) were chosen for each PZA monoresistant case (Figure 1). PZA monoresistance was associated with extrapulmonary disease compared to drug susceptible controls both in national surveillance data (aPR of 3.09) [1] and in our study population ([OR], 6.0; 95% [CI], 2.70–13.26) (Table 1). Controls were therefore matched to cases with the same site of infection, either extrapulmonary or pulmonary, in order to avoid differences in affected sites as a confounding variable. In the second case-control group patients infected with MDR and PZA resistant *M.tb* (PZA-MDR; *n* = 17) were compared to a control group composed of all those infected with MDR *M.tb* (*n* = 25; Figure 1).

**Table 1:** Site of infection based on tuberculosis drug resistance pattern in San Francisco between 1991 and 2011.

Site of infection	PZA monoresistant (*n* = 25)	Pan-susceptible (*n* = 2501)		MDR-PZA (*n* = 17)	MDR (*n* = 25)	
	*n* (%)	*n* (%)	*p*	*n* (%)	*n* (%)	*p*
Pulmonary	11 (44)^a^	1991 (80)	Ref	14 (82)	21 (84)	Ref
Pulmonary & extrapulmonary	0	86 (3)	b	0	3 (12)	b
Extrapulmonary	14 (56)^a^	424 (17)	<0.001	3 (18)	1 (4)	0.21

a Five of the pulmonary and seven of the extrapulmonary infections were from *M. bovis*.

b *p* values for multinomial logistical regression analysis are not recorded for categories with zero cases.

INH, RIF, and EMB drug sensitivity was determined using the agar proportion method, Bactec 460, or MGIT 960 systems in years 1991–1994, 1994–2007, or 2007–2011, respectively. PZA resistance testing was performed using liquid media in the Bactec 460 or MGIT 960 system at a PZA concentration of 100 µg/ml.

Genotyping of *M.tb* was performed using restriction fragment length polymorphisms with the insertion sequences (IS) *6110* [8] and polymorphic guanine-cytosine rich sequencing (PGRS) [9]. The phylogeographic lineage of the *M.tb* isolates was determined using regions of difference (RD) and single nucleotide polymorphisms [5].

Treatment failure was defined as culture positivity four months after treatment [10]. Mortality rates included deaths within six months of tuberculosis diagnosis [3]. Disease recurrence was defined as a second episode of tuberculosis within one year of completing therapy [10]. The combined treatment outcome included treatment failure, mortality, and recurrence. Clusters of *M.tb* were defined as two or more persons having *M.tb* isolates with the same IS*6110-* and PGRS (if fewer than six IS*6110* bands) [11] and within a two year time frame [12].

### Statistical analysis

Stata/SE 12.1 was used for statistical analysis. Bivariate unadjusted analysis was performed with the chi-squared test for dichotomous variables. The single continuous variable, treatment length, was not normally distributed and was analyzed using the Wilcoxon Rank-sum (Mann-Whitney) test. Multivariate logistic regression analysis was performed including predictor variables associated with *p*<0.2 in bivariate analysis. A *p* value of <0.05 was considered significant. Multivariate analysis was performed with race, HIV, and prior *M.tb* diagnosis or treatment as predictor variables. The infection site, extrapulmonary alone, was also included as a predictor variable in the analysis of the MDR and PZA-MDR comparison cohort.

## Results

Between January 1991 and December 2011, PZA resistance testing was performed on baseline samples from 2,842 culture confirmed TB cases in San Francisco (Figure 1). De novo PZA resistance was identified in 1.8% of cases (50 of 2,842; Figure 1 and Table 2). Of the 50 PZA resistant cases, 25 isolates were PZA monoresistant (Figure 1). There were 25 MDR TB cases during the study period as well as 17 additional cases of PZA-MDR infection (Figure 1). Thus, 40% of MDR isolates were also PZA resistant. There were eight non-MDR cases resistant to PZA and other antituberculous medications (Figure 1). PZA monoresistant cases were distributed evenly in time across the 21 year study period, with an average of 1.2 cases being identified per year, corresponding to a case rate of 0.3 per 100,000. We identified two clusters of *M.tb* disease with matching restriction fragment length polymorphism patterns, totaling four cases. One of the clusters was a transmission chain involving two MDR cases in the year 1992. The other cluster was a transmission chain involving two *M. bovis* cases in the years 1995 and 1997.

**Table 2:** Characteristics of patients with drug susceptible, PZA resistant, and MDR mycobacterial infection in San Francisco between 1991 and 2011.

Characteristic	PZA monoresistant (*n* = 25)	Pan-susceptible controls (*n* = 75)		MDR-PZA (*n* = 17)	MDR (*n* = 25)	
	*n* (%)	*n* (%)	*p*	*n* (%)	*n* (%)	*p*
Male	16 (64)	45 (60)	.72	6 (35)	12 (48)	.42
Age≥65 years	10 (40)	23 (31)	.39	1 (6)	3 (12)	.52
Ethnicity^a^						
Non-hispanic white	4 (16)	12 (16)	.26	3 (18)	7 (28)	.42
Non-hispanic black	3 (12)	8 (11)	.24	0	1 (4)	.99
Hispanic	12 (48)	13 (18)	.002	1 (6)	1 (4)	.89
Asian/Pacific Islander	6 (24)	41 (55)	Ref	13 (76)	16 (64)	Ref
Foreign-born	16 (64)	54 (72)	.45	15 (88)	20 (80)	.49
HIV-infection						
Positive	4 (16)	13 (17)	.91	1 (6)	3 (12)	Ref
Negative	10 (40)	30 (40)	Ref	7 (41)	16 (64)	.83
Unknown	11 (44)	32 (43)	.95	9 (53)	6 (24)	.08
Homeless	2 (8)^b^	10 (13)^b^	.47	0	0	1
Drug or Alcohol Abuse	5 (20)	11 (15)	.62	2 (12)	3 (12)	.98
Prior diagnosis of *M.tb*	3 (12)	4 (6)	.31	11 (65)	8 (32)	.04
AFB smear positive	5 (22)	18 (25)	.73	7 (44)	12 (48)	.79
Cavitary chest X-ray^c^	1 (4)	8 (11)	.33	5 (29)	9 (36)	.76
Extrapulmonary only	14 (56)	42 (56)	1.0	3 (18)	1 (4)	.21
Completed therapy	22 (88)	62 (83)	.53	13 (76)	19 (83)	.63
PZA included in the treatment^d^	18 (75)	59 (83)	.38	15 (100)	18 (100)	1
**Treatment outcomes**						
Combined outcome	1 (4)	6 (8)	.51	3 (18)	7 (29)	.40
Mortality	0 (0)	6 (8)	e	2 (12)	1 (4)	.38
Treatment failure	1 (4)	0	e	1 (6)	6 (25)	.14
Treatment median, mos.	9.7	8.3	.008	19.0	19.7	.56
**Lineage**						
Indo-Oceanic	1 (4)	14 (19)	.32	2 (12)	8 (32)	.16
Euro-American	7 (28)	32 (43)	Ref	6 (35)	6 (24)	Ref
East Asian	2 (8)	12 (16)	.76	5 (29)	8 (32)	.56
Unknown	3 (12)	17 (23)	.78	4 (24)	3 (12)	.76
***M. bovis***	12 (48)	0	e	0	0	e

Abbreviations: HIV, Human Immunodeficiency Virus; AFB, acid-fast bacilli; Ref, referent group.

a One of the susceptible cases was Native-American.

b Housing status was not known for one of the patients.

c X-ray data was not available for one of the susceptible controls and one of the PZA-MDR cases.

d Three of the susceptible cases died upon diagnosis and one moved. One of the PZA monoresistant cases died before treatment. One of the MDR cases was lost to follow up. Treatment regimens were not available for 5 of the PZA monoresistant, 7 of the MDR, and 2 of the PZA-MDR cases. These cases were excluded from treatment outcomes analysis.

e *p* values for multinomial logistical regression analysis are not recorded for categories with zero cases.

The two patient characteristics significantly associated with PZA monoresistance were Hispanic race ([OR], 6.3; 95% [CI], 1.97–20.16) and extrapulmonary disease ([OR], 6.0; 95% [CI], 2.70–13.26) (Tables 1 and 2). The majority of PZA monoresistant cases in Hispanics were from *M. bovis* (67%; *n* = 8). There was no association between PZA monoresistance and HIV serostatus. There were no significant differences in patient characteristics between between PZA-MDR and MDR groups. Genotype analysis revealed no significant differences in the distribution of *M.tb* lineage in PZA monoresistant or PZA-MDR groups compared to drug susceptible and MDR cases, respectively.

There were no significant differences in the percentage of patients treated with PZA during the intensive phase of treatment between the comparison groups of drug susceptible and PZA monoresistant cases (83% vs. 75%, *p* = 0.38). All the patients with MDR and PZA-MDR *M.tb* were treated with PZA, but treatment regimen data was not available for seven and two of the cases, respectively. The potential impact of PZA treatment in these groups must therefore be interpreted with caution. Median treatment duration was significantly longer in the PZA monoresistant cases (9.7 months) compared to the susceptible controls (8.3 months; *p* = 0.008).

In bivariate analysis cases with PZA resistance had decreased rates of the combined treatment outcome when comparing PZA monoresistant cases to drug susceptible *M.tb* (4% vs. 8%, *p* = 0.51), and when comparing PZA-MDR to MDR cases (18% vs. 29%, *p* = 0.40), but neither of these differences were statistically significant. None of the patients experienced a recurrence of disease. In multivariate analysis the combined treatment outcome was not different between PZA monoresistant and drug susceptible cases ([OR], 0.27; [CI], 0.02–2.96) or MDR-PZA compared to MDR cases ([OR], 0.31; [CI], 0.04–2. 12).

## Discussion

PZA resistance was identified in 1.8% (50 of 2,842) of mycobacterial isolates tested in San Francisco between 1991 and 2011. This observation is consistent with 2–3.3% rate of PZA resistance among clinical isolates reported in national surveillance data [1]. In contrast, the relatively higher PZA resistance rate of 6% reported in the Canadian province of Quebec was in part attributed to an ancestral mutation in the *pnc*A allele that persisted in contemporary strains [3]. PZA resistance rates in San Francisco were lower than the national INH resistance rate of 6.1%, but higher than the MDR rate of 1.1% between years 1993 and 2011 [13].

Our cohort analysis found that illness from *M. bovis* predominantly occurs in Hispanics and is associated with extrapulmonary disease. Our study is in agreement with published reports demonstrating association between birth in Mexico and *M. bovis* infection in San Francisco and San Diego [14]. In addition, infection with *M. bovis* [15] and PZA monoresistant *M.tb* [1] were associated with both extrapulmonary infection and Hispanic ethnicity in analyses of the US NTSS. Extrapulmonary disease from *M. bovis* in Hispanics is thought to be from foodborne exposure of unpasteurized, contaminated dairy products from Mexico [15]. *M. bovis* infection in the Hispanic immigrant population from Mexico highlights a community that may particularly benefit from mycobacterial speciation or PZA resistance testing.

PZA resistance was identified in 38% of MDR cases in national surveillance data [1]. Similarly, we found that 40% (17 of 42) of patients in San Francisco and 45% (13 of 29) of Asian-Pacific patients infected with MDR *M.tb* were also PZA resistant (Table 2). These results emphasize the importance of PZA resistance testing particularly among those with MDR TB and Asia-Pacific ethnicity.

In contrast to the study from Quebec, we did not find a statistically significant association between PZA resistance and treatment failure; however, in our study patients with PZA monoresistance received significantly longer treatment than drug-susceptible *M.tb* cases (9.7 vs. 8.3 months, *p* = 0.008) unlike the study in Quebec. Modification of treatment regimens once PZA resistance data was available at the end of the intensive phase of treatment may in part explain why there were no differences in treatment outcomes in our study. Unlike the NTSS study, we did not find an association between PZA resistance and lineage. Although we analyzed every case of PZA resistant infection in an otherwise drug susceptible or MDR background in San Francisco County, our study was underpowered to make definitive conclusions about the impact of PZA resistance on treatment outcomes.

Additional limitations of our study are that BCG (Bacille Calmette-Guérin) vaccination history was not available, and genotyping was not performed for the identification of the *M. bovis* BCG strain. Disease from BCG can uncommonly occur in recently vaccinated immunocompromised children co-infected with HIV [16] or in adults with bladder cancer treated with intravesicular BCG [17]. However, infection with *M. bovis* BCG is rare in the USA and was identified in 0.4%, or 10 isolates, of 14,157 tuberculosis samples submitted to the NTSS [15].

In sum, across 21 years of programmatic and molecular data from San Francisco, our findings are reassuring in that PZA resistance was uncommon and its presence did not significantly impact treatment outcome rates compared to susceptible controls or MDR controls, albeit with extended treatment duration as recommended by national TB treatment guidelines [10]. Additional research will be needed to determine definitively whether novel shortened regimens that rely on PZA as a key component will be adversely impacted from PZA monoresistance.
